# Speciation Analysis of Trace Arsenic, Mercury, Selenium and Antimony in Environmental and Biological Samples Based on Hyphenated Techniques

**DOI:** 10.3390/molecules24050926

**Published:** 2019-03-07

**Authors:** Xiaoping Yu, Chenglong Liu, Yafei Guo, Tianlong Deng

**Affiliations:** Tianjin Key Laboratory of Marine Resources and Chemistry, College of Chemical Engineering and Materials Science, Tianjin University of Science & Technology, Tianjin 300457, China; 17302297916@163.com (C.L.); guoyafei@tust.edu.cn (Y.G.)

**Keywords:** arsenic, mercury, selenium, antimony, speciation analysis, hyphenated technique

## Abstract

In order to obtain a well understanding of the toxicity and ecological effects of trace elements in the environment, it is necessary to determine not only the total amount, but also their existing species. Speciation analysis has become increasingly important in making risk assessments of toxic elements since the toxicity and bioavailability strongly depend on their chemical forms. Effective separation of different species in combination with highly sensitive detectors to quantify these particular species is indispensable to meet this requirement. In this paper, we present the recent progresses on the speciation analysis of trace arsenic, mercury, selenium and antimony in environmental and biological samples with an emphasis on the separation and detection techniques, especially the recent applications of high performance liquid chromatography (HPLC) hyphenated to atomic spectrometry or mass spectrometry.

## 1. Introduction

Trace elements, as important components of the environment, play crucial roles in the functioning of life. Some elements such as arsenic (As), mercury (Hg) and antimony (Sb) can be highly toxic to various life forms, while some others are probably considered to be essential, but they can become toxic at higher doses. For example, selenium (Se) is widely recognized as an essential dietary component with numerous beneficial effects on health, but at levels higher than 5 µg/L will cause toxic effects [1,2]. It is generally recognized that the mobility, toxicity and bioavailability of trace elements depend strongly on their particular existing forms, and the determination of species rather than the total amount of an element is more important [3]. Usually, the ecological and healthy risk assessments of trace elements in the environment based on the species data will seem to be more reasonable and accurate than the total amount data.

“Chemical species” refers to the specific form of an element defined as to isotopic composition, electronic or oxidation state, and/or complex or molecular structure [4]. Usually, compounds that differ in isotopic composition, conformation, oxidation or electronic state, or in the nature of their complexed or covalently bound substituents, can be regarded as distinct chemical species [5]. The division suggested by Tessier et al. [6] is usually recommended in the research relevant to the species of heavy metals in soil or sediment. They distinguished and defined five fractions, i.e., exchangeable, carbonate-bound, iron and manganese oxides-bound, organic matter-bound, and the other mineral-bound metals. In addition, the BCR or modified BCR sequential extraction method [7,8] and the modified Tessier method [9] are also used to study the bound species of heavy metals in soil or sediment [10]. Nonetheless, these methods do not allow the differentiation between oxidation states of elements in aqueous phase, of which may be of great importance when considering their toxicity. As, Hg, Sb and Se probably exist in many forms in environmental and biological samples as shown in Table 1. 

However, the main species existing in aquatic environments are inorganic forms, i.e., As(III), As(V); Hg(II); Sb(III), Sb(V); and Se(IV), Se(VI) [11,12,13], but methylation may occur in sediment environments due to the actions of microorganisms [14,15]. Selenosugars have been confirmed to be important urinary selenium metabolites, while selenohomolanthionine (SeHLan) is mainly detected in some Se-accumulating plants and yeasts [16,17,18]. Lipid-soluble arsenic compounds (arsenolipids) are mainly found in fish oils, fish liver, sashimi tuna, algae, et al. [19,20,21]. Of course, some unusual chemical species in biosamples or other complex samples probably exist. For example, tetramethylarsonium ion (TETRA), glyceryl phosphorylarsenocholine (GPAsC), and dimethyl- arsinothioic acid (DMAS) were found and identified in marine foods [22]; 4-aminophenylarsonic acid (4-APAA) and *N*-acetyl-4-hydroxyphenylarsonic acid (*N*-AHPAA) were found in chicken liver [23].

The lack of accurate speciation information is the major limitation for us to understand the environmental biogeochemical cycles of trace elements in the aquatic environments. In view of this, speciation analysis has become one of the fastest developed areas of analytical chemistry over the last two decades. “Speciation analysis” is defined by the IUPAC as analytical activities of identifying and/or measuring the quantities of one or more individual chemical species in a sample. It plays a unique role in the studies of biogeochemical cycles of compounds, determination of toxicity and eco-toxicity of selected elements, quality control of food products, control of medicines and pharmaceutical products, examination of occupational exposure and clinical analysis, etc. [24]. Generally, two complementary techniques are necessary for the speciation analysis of trace elements, i.e., separation and detection. The former provides an efficient and reliable separation of the species, and the latter provides adequate detection and quantification. Progresses in analytical instruments and methodology allow us to identify and measure the species presented in a particular system. Especially, the coupling of chromatographic techniques, such as gas chromatography (GC) and high performance liquid chromatography (HPLC), with a highly sensitive and selective detector, such as mass spectrometry (MS) or atomic spectrometry including atomic fluorescence spectrometry (AFS), atomic absorption spectrometry (AAS), and atomic emission spectrometry (AES) or optical emission spectrometry (OES) has been widely exploited and accepted for the speciation analysis of trace elements. Even though several reviews have been dedicated to the speciation analysis of As, Hg, Se, and Sb [25,26,27,28,29,30,31,32], this presented work reviews and discusses the different separation and detection methods for the speciation analysis of trace As, Hg, Se and Sb in environmental and biological samples with an emphasis on the hyphenated techniques.

## 2. Separation Techniques for Speciation Analysis

### 2.1. Non-Chromatographic Methods

The chemical form and quantitative information of a given element can be acquired by the application of basic chemistry methods. In other word, non-chromatographic procedures can provide simple methods to obtain sufficient information on the elemental species. Many review papers are available for the non-chromatographic speciation analysis methods [33,34,35], and the commonly used for As, Hg, Se and Sb include extraction and selective reduction. 

#### 2.1.1. Extraction

Extraction is usually used to separate one or a group of species from complex matrices, especially for environmental and biological samples [36,37]. Liquid-liquid extraction (LLE) is the oldest pre-concentration and isolation method for speciation analysis, and can be directly applied for the non-filtered samples with complex matrices [38]. Sequential extraction procedures using aqueous solutions, such as the Tessier or BCR method, are usually used to characterize the mobility, bioavailability, and potential toxicity of trace elements in soil and sediment. LLE may perform best for the redox labile elements such as As, Sb and Se, for which more discrete biogeochemical species may be generated due to the variations in oxidation number. At present, LLE is rarely applied for elemental species in water samples except for the extraction of different bounded species in sediment or soil. Some modified LLE methods are also used for speciation analysis. For example, ultrasonic assisted dispersive liquid-liquid microextraction (LLME) was used by Panhwar et al. [39] to analyze Se(IV) and Se(VI) in water and food samples. A vortex assisted dispersive liquid-liquid microextraction method based on the freezing of deep eutectic solvent was developed by Akramipour et al. [40] for the determination of organic and inorganic mercury in blood samples. Based on the principles of extraction, some other extracting methods have been developed including liquid phase microextraction (LPME) [41,42], solid phase extraction (SPE) [43,44], solid phase microextraction (SPME) [45,46,47,48], etc. 

LPME is introduced to reduce the consumption of solvent, in which a drop of organic solvent is suspended at the tip of a microsyringe and exposed to the analytical sample. Subsequently, the drop is retracted and transferred to a particular analytical instrument after extraction. For example, speciation analysis of As(III) and As(V) based on Triton X-100 hollow fiber LPME coupled with flame atomic absorption spectrometry (FAAS) was developed by Zeng et al. [42], during which the Triton X-100 was used as an extractant and an acceptor solution; Fan [49] determined Sb(III) and total Sb in natural water by electrothermal atomic absorption spectrometry (ET-AAS) prior to separate and preconcentrate using *N*-benzoyl-*N*-phenylhydroxylamine-chloroform single drop; Single-drop gold nanoparticles for headspace microextraction and colorimetric assay of Hg(II) in environmental waters was developed by Tolessa et al. [50], and the recovery for a 10 nM spiked level was in the range of 86.8~99.8%.

SPE is popular for sample preparation in organic analysis, but it is also found to be used for speciation analysis of inorganic elements. The analytes are extracted by selective sorption and subsequently derivatized or directly detected after eluted with a small amount of organic solvent [51,52]. Sorbents with an immobilized chelating reagent such as ammonium pyrrolidine dithiocarbamate (APDC) are widely used in SPE. For example, a new magnetic SPE using octyl-immobilized silica-coated magnetic Fe_3_O_4_ (C_8_-Fe_3_O_4_@SiO_2_) nanoparticles was proposed by Li et al. [53] for the determination of trace Sb(III) and Sb(V) in water, during which Sb(III) forms a hydrophobic complex with APDC at pH 5.0 and is retained on C_8_-Fe_3_O_4_@SiO_2_ nanoparticles, whereas Sb(V) remains as free species in aqueous solution. A method for multi-element inorganic speciation analysis of As(III, V), Se(IV, VI) and Sb(III, V) in natural water using SPE technology was developed by Zhang et al. [54], during which TiO_2_ was used to adsorb total inorganic As, Se and Sb, while As(III), Se(IV) and Sb(III) were coprecipitated with Pb-PDC. The concentration of As(V), Se(VI) and Sb(V) was subsequently calculated by the differences. Recently, Peng et al. [55] synthesized and employed functionalized multi-wall carbon nanotubes as the adsorbent for simultaneous speciation analysis of inorganic As, Se and chromium (Cr) in environmental waters by microcolumn SPE. The detection limits of 15, 38 and 16 ng/L were obtained for As(V), Cr(VI) and Se(VI), respectively.

SPME offers a fast way for species separation by collecting target analytes from the sample headspace or liquid phase directly or after derivatization. It offers the possibility to eliminate the interferences from matrices without the use of organic solvents [44,56,57]. For example, headspace SPME coupled to miniaturized microplasma OES was developed by Zheng et al. [57] for the detection of mercury and lead in water samples. It is noted that SPME is also often used as a pretreatment method to separate or preconcentrate analytes prior to chromatographic separation [58,59]. Of course, direct coupling of SPME with particular detectors is also developed. For example, SPME was used by Panhwar et al. [45] for the determination of inorganic Sb speciation. Sb(III) forms hydrophobic complex with diethyl dithiocarbamate at pH 5.5 and subsequently adsorbed on polystyrene oleic acid imidazole polymer. A screening method was developed by Mester [60] to determine volatile metallic compounds (As, Se, Sn and Sb) in solid samples by examining the vapor phase above the sample with a SPME probe. The total analysis time was less than three minutes depending on the concentration of the target compounds.

Some assistant extracting techniques such as microwave-assisted extraction (MWAE) and ultrasound-assisted extraction (USAE) [39,61,62,63,64] have also been applied for speciation analysis so as to improve the speed of extraction. It is noted that although extraction methods can realize the determination of elemental speciation, they are usually used as pretreatment procedures for elemental speciation in complex samples followed by the chromatographic hyphenated techniques for species separation and detection [65,66,67]. For example, in order to determine As speciation in guano and ornithogenic sediments, MWAE was used by Lou et al. [65] to extract As(III), DMA, MMA, and As(V) in these sediments followed by the detection of HPLC-HG-AFS.

#### 2.1.2. Selective Reduction

Selective reduction is used based on the differences of reduction potential between different species. The reduction potential can be controlled by the concentration of reductants, pH, as well as by the presence of catalysts or chelating agents. Selective reduction is generally related to the chemical vapor generation (CVG) methods, by which the volatile derivatives are produced during reduction [68,69]. 

It is noted that some organic Se and Hg species are not easily reduced by BH_4_^−^, which is the most frequently used reductant during CVG. In view of this, photochemical reduction by exposure to ultraviolet (UV) irradiation or ultrasound (US) is often used. For example, when Hg(II) and MeHg were determined by Hu et al. [70] using hydride generation ultraviolet atomization-AFS, the Hg(II) can be directly measured under the non-ultraviolet radiation mode after reducing using 0.1% (*m*/*v*) KBH_4_, while the MeHg needs to be transformed into elemental mercury vapor under the ultraviolet atomization. when total Hg and MeHg in biological samples were detected by Vieira et al. [71], total Hg was measured after the tissues were digested in either formic acid or tetramethylammonium hydroxide (TMAH) following the reduction of both species by exposure the solution to UV irradiation, during which MeHg was selectively quantitated by adding of 10% *v*/*v* acetic acid into TMAH solution. Mendez et al. [72] assessed the UV and US induced redox reactions for the determination of Se(IV), Se(VI), SeMet, and SeCys in model water, enriched natural water, and soil/fly ash extracts using HG-AAS and AFS. Furthermore, Chen et al. [73] systematically studied the photochemical behaviors of selenium and some of its organic compounds in various aqueous matrices under UV irradiation at 300 nm. It was observed that the photochemical oxidation rate of Se(IV) to Se(VI) was greatly enhanced in the presence of HNO_3_ at concentration larger than 1×10^−3^ M. Subsequently, the same authors [74] developed a method for the speciation analysis of Se in natural waters based on the photochemical reactions of Se(IV) and organic selenium in different aqueous solutions. 

#### 2.1.3. Miscellaneous Methods

Due to the different volatility of Hg and its compounds, thermal desorption is also used to distinguish their different species. For example, thermal release analysis in combination with AAS was developed and applied by Shuvaeva et al. [75] to determine Hg(II), MeHg, and mercury sulfide in lake sediment and plankton. Similar technique was also used by Kaercher et al. [76] for the determination of inorganic and total Hg in biological samples based on the temperature control of the measurement cell. Masking with relevant masking agents is sometimes used for speciation analysis. For example, 8-hydroxyquinoline was used as an effective masking agent by Liao and Deng [77] for As speciation analysis in porewater and sediment. In addition, in order to realize redox speciation analysis of Sb in water, Xi et al. [78] systematically tested several compounds as masking agents to inhibit the generation of stibine from Sb(V), and the results indicated that citric acid and NaF can successfully suppress this process.

It is noted that any pretreatment processes during speciation analysis even the routine processes such as the changes of pH, temperature and pressure can bring about irreversible transformation of the analyzed species. Non-chromatographic speciation analysis methods often employ multi-step procedures resulting in a high risk for the loss of analytes or species conversion. In this sense, these characteristics make such methods very difficult to automate and integrate into modern systems. Therefore, high selective and sensitive analytical methods without or with few pretreatment steps are more competent for speciation analysis. 

### 2.2. Chromatographic Hyphenated Techniques

The most detailed information in relation to speciation analysis is no doubt derived from hyphenated techniques, in particular those involving separation by HPLC, GC or CE with ICP-MS or atomic spectrometry detectors. The main advantages of those techniques include extremely low detection limit, insignificant interference, high precision and repeatability, etc. The selection of a proper separation technique depends on the physico-chemical properties such as volatility, charge or polarity of the different species, and sometimes combination of two or more separation methods is also adopted.

#### 2.2.1. Gas Chromatography

GC is mainly used for Hg species separation when compared with that used for As, Se and Sb, during which volatile Hg species are stripped from the sample solution after derivatization, and subsequently pre-concentrated mainly through sorptive, extraction, etc [79,80,81]. The trapped Hg derivatives are then released thermally and transferred quantitatively to the GC for species separation. 

Because the packed columns will lead to poor reproducibility, thus capillary or multi-capillary GC is usually used to provide superior separation power and better detection limits [82]. Usually, the Hg derivatives need to be quantitatively transferred to a suitable detection system after the GC separation. A comparative study of GC coupled with AFS, AES and MS for MeHg and EtHg analysis following aqueous derivatization was conducted by Cai et al. [83], and found that both GC-AFS and GC-AES shown to be excellent techniques with detection limits in the range of sub-picogram levels. Nevado et al. [84] also evaluated the advantages and disadvantages of three hyphenated techniques including GC-MS, GC-ICP-MS and GC-pyrolysis-AFS for Hg speciation analysis in different sample matrices after aqueous ethylation with sodium tetraethylborate. Absolute detection and quantification limits were in the range of 1~4 pg for GC-MS, 0.05~0.21 pg for GC-ICP-MS, and 2~6 pg for GC-pyro-AFS.

Of course, there are also some applications of GC on the speciation analysis of As, Se and Sb [85,86,87,88,89]. For example, volatile organic Se compounds of dimethylselenide (DMSe) and dimethyldiselenide (DMDSe) in environmental and biological samples were determined by Ghasemi et al. [88] using a headspace hollow fiber protected LPME combined with capillary GC-MS. It is noted that although GC attracts particular attention due to its high efficiency and simplicity of coupling, however, in contrast to GC, HPLC has the ability of dealing with non-volatile compounds without any derivatization in some cases, and thus extending the range of application.

#### 2.2.2. Liquid Chromatography

Among the most popular hyphenated techniques used for the determination of As, Hg, Se and Sb species, the coupling of various liquid chromatographies (LC) including ion-exchange chromatography (IEC) and reverse-phase chromatography (RPC) with particular detectors has obtained the fastest development [90]. Table 2 shows the recent applications of hyphenated techniques based on LC separation for the speciation analysis of As, Hg, Sb and Se in environmental and biological samples.

**Figure 1:**
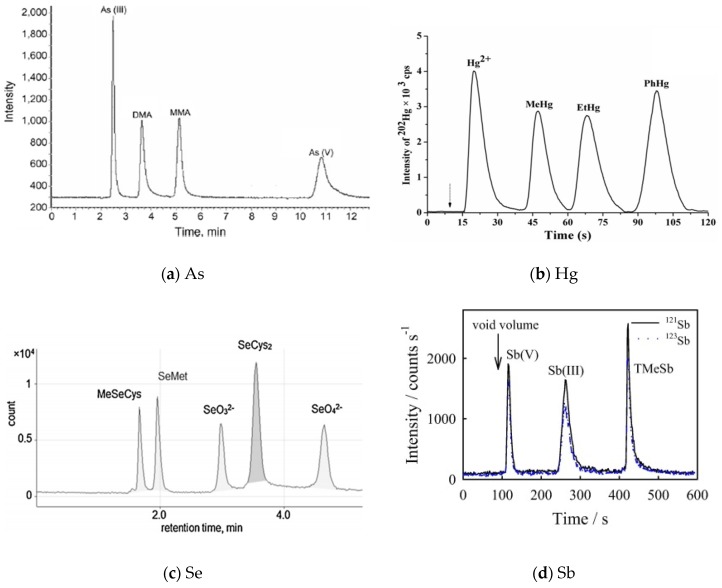
Representative chromatograms of As, Hg, Se, and Sb species. (**a**) As: each at 100 μg/L. Detected by HG-AFS after separated by a Hamilton PRP-X100 column (250 mm × 4.1 mm i.d., 10 μm) and eluted using 15 mM (NH_4_)_2_HPO_4_ (pH 6.0) at 1.0 mL/min flow rate [127]; (**b**) Hg: each at 5.0 μg/L. Detected by ICP-MS after separated by two consecutive Zorbax SCX columns (12.5 mm × 4.6 mm i.d., 5 μm) and eluted by 2.0 mM thiourea (pH 2.0) at 1.5 mL/min flow rate [128]; (**c**) Se: Detected by ICP-MS after separated by a Dinoex IonPac AS11 anion exchange column (4 mm i.d. × 250 mm) and eluted using 10 mM NaHCO_3_ with 2% acetonitrile (pH 11 adjusted with 20% NH_3_) at 0.6 mL/min flow rate [129]; (**d**) Sb: 1 μg/L Sb(V), 2 μg/L Sb(III) and TMeSb. Detected by ICP-MS after separated by a Hamilton PRP-X100 column (250 × 4.1 mm i.d., 10 μm) and eluted using A: 20 mM EDTA + 2 mM KHP (pH 5.5), B: 20 mM + 2 mM KHP + 40 mM (NH_4_)_2_CO_3_ + 1% (*v*/*v*) CH_3_OH (pH 9.0) at 1.2 mL/min flow rate [123].

The separation of species in IEC is based on the interactions between the positively or negatively charged species and the stationary phase that contains a cationic functional group (anion exchange) or an anionic functional group (cation exchange). IEC is an ideal technique for the separation of inorganic As, Sb, Se and many charged organometallic ions such as organoselenium and organoarsenic. Buffer solutions are commonly used as eluents for IEC with concentration usually not exceeding 25 mM. RPC is used based on the partition of the analytes between a non-polar stationary phase, in which it usually contains a covalently bound C_8_ or C_18_ linear hydrocarbon, and a relatively polar mobile phase. RPC is usually superior to IEC for the separation of organometallic species. Figure 1 shows the representative chromatograms of As [127], Hg [128], Se [129], and Sb [123] species in aqueous solutions separated by IEC or RPC.

IEC is the most extensively used method for As, Sb and Se speciation separation, followed closely by the use of ion-pair reversed-phase chromatography (IP-RPC). Many factors, such as the pH, ionic strength, concentration and flow rate of mobile phase, the concentration and species of organic modifiers, will all influence the separation and retention of analytes in IEC and IP-RPC. For example, arsenic species are neutral, anionic or cationic depending on the pH. As a result, their retention will strongly depend on the pH of mobile phase. Therefore, anion-exchange chromatography is often used to separate As(III), As(V), MMA and DMA, while cation-exchange chromatography is used to separate AsB, AsC, TMAO, Me_4_As^+^ for such compounds at cationic or neutral states cannot be retained by an anion-exchange mechanism. Of course, both anion and cation exchange columns are sometimes used in a complementary fashion [130]. Based on the requirement of pH, phosphate [131], carbonate [132], nitrate [133], acetate [134] or miscellaneous [122,135] are commonly used as buffers. The anion-exchange mode with phosphate buffer elution is classically used for the separation of As(III), As(V), MMA and DMA. Sodium salt buffer leaves carbon residue upon the sampler and skimmer cones of ICP-MS resulting in the instability of plasma and the shift of retention time. Therefore, mobile phases based on ammonium salts are often used. For example, the use of NH_4_NO_3_ as mobile phase was shown to produce good signal stability on the ICP-MS with minimal salt deposit on the sample and skimmer cones [136].

When IP-RPC is used for species separation, a counterion is added to the mobile phase, and a secondary chemical equilibrium of the ion-pair is used to control the retention and selectivity [115,119,137]. The resolutions of species depend on the concentration and kind of ion-pair reagents, organic modifiers (e.g., methanol or acetonitrile), ionic strength, and pH of the mobile phase, etc [138]. The most used ion-pair reagent is tetrabutylammonium (TBA, including hydroxide, phosphate and bromide). Le et al. [139] investigated a series of ion pair reagents having different strength such as methanesulfonate, ethanesulfonate, propanesulfonate for the simultaneous analysis of Se and As species in a reversed phase C18 column, and found that all seven As species investigated were well separated within 16 min using hexanesulfonate as ion pair reagent. Choosing a suitable pH of eluent is also critical for species separation by IP-RPC. For example, As(III) is a neutral species, and will be eluted in the void volume at a low pH of mobile phase. But it becomes a negatively charged species when the pH of mobile phase is increased above its pK_a_ value of 9.2. Chromatographic column also plays an important role in the separation of species. Usually, different chromatographic columns of the same separation mechanism have different separation effects. For example, Ammann [140] investigated two different polymeric anion-exchangers: a low capacity, weakly hydrophobic material (AS11, Dionex) and a more frequently used higher capacity, higher hydrophobicmaterial (AS7, Dionex), and found that AS11 provided better retention for MMA, AsB, As(III) than AS7, whereas DMA and Cl^−^ were more retained on AG7. Gao et al. [119] compared the separation effects of five selenium species by two anion-exchange columns (Hamilton PRP X-100 and Dionex AS19) and three typical reversed C18 columns (Agilent Eclipse Plus C18, Waters Xselect HSS T3 and StableBond C18), and found that the StableBond C18 is more robust or has a better resolution.

When LC is employed for Hg species separation, a RP column is commonly used. However, the organic solvent concentration in the mobile phase must be as low as possible in order to reduce carbon deposit on the ICP-MS instrument interface. In order to overcome the drawback caused by organic solvent, vapor generation (VG) technique can be employed after the LC separation, by which only the volatile Hg species are introduced into the plasma. Of course, Hg(II), MeHg, EtHg and PhHg can be separated in a cation-exchange chromatographic column due to they present in positively charged ions. For example, the four species were separated and determined by Chen et al. [128] by the coupling of cation-exchange chromatographic separation with ICP-MS detection. 

Both isocratic and gradient ion-exchange chromatographic systems are used for species separation. Gradient separation generally has better resolution among different species, and is often used to reduce analytical time of strong retention species. However, gradient IP-RPC is not commonly adopted when ICP-MS is used as detector due to the signal drift is likely when substantially changing the organic content of the mobile phase. In addition, as the differences in structure and charge of different species, a single chromatographic mechanism is probably not sufficient for simultaneous study of organic and inorganic species. Combination of different chromatographic modes has therefore been applied by using columns in series or column-switching systems. For example, Milstein et al. [141] successfully separated and determined As(III), As(V), MMA, DMA, AsB, and AsC by connecting cation- and anion-exchange columns in series and eluting by (NH_4_)_2_CO_3_ buffer. Of course, it is noted that the mobile phase used in multidimensional chromatography should be compatible with both chromatographic mechanisms.

#### 2.2.3. Capillary Electrophoresis

Capillary electrophoresis (CE) is discussed as a complementary technique to GC and HPLC, and is a powerful tool in element speciation with high separation capability and environmentally friendly nature due to the use of aqueous buffer solutions with moderate pH and its extremely low reagent and sample consumption [142]. For example, ten As compounds including As(III), As(V), MMA, DMA, AsB, AsC, Rox, *o*-arsanilic acid, *p*-ureidophenylarsonic acid, and 4-nitrophenylarsonic acid were simultaneously determined by Liu et al. [143] by CE coupled with ICP-MS. Of course, for the analysis of elements in complex matrices, some pretreatment steps such as microextraction techniques are often necessary prior to CE separation [144,145,146].

Separation by CE is usually faster than that by LC, and therefore is potentially a rapid and highly efficient separation technique. However, some problems are presented when CE is coupled to ICP-OES or ICP-MS due to liquid flow incompatibility, i.e., the liquid flow is one order of magnitude lower in CE than in ICP sample introduction system. The detection limit is often insufficient resulting from the very small sample volumes used in CE, and therefore some interface techniques were developed. For example, a novel system for CE and ICP sample introduction that incorporates a dedicated Flow Focusing^®^ based nebulizer as aerosol generation unit was presented by Kovachev et al. [147], and on-line coupling of CE with ICP-MS was developed by Liu et al. [148] using a sprayer with a novel direct-injection high-efficiency nebulizer (DIHEN) chamber as the interface. HG technique is also often integrated into CE hyphenated systems to provide a kind interface method. For example, a microfluidic chip-based capillary electrophoresis (μchip-CE) HG system was interfaced with a microwave induced plasma optical emission spectrometry (MIP-OES) by Matusiewicz and Ślachciński [149] to provide As(III) and As(V) species separation capabilities. Although CE provides an effective measure to fulfill species separation, there still exist major challenges that limit its practical acceptance. For example, no sufficient care on possible changes in speciation during electrophoresis, no appropriate treatment on method validation and system suitability aspects, etc [150].

It is noted that although there has been significant progress in speciation analysis based on the hyphenated chromatographic separation with atomic spectrometry or mass spectrometry detectors, and these hyphenated methods can provide the most complete information on the species distributions and even structures, they also have themselves disadvantages. A limitation related to the hyphenated techniques is the low sample volume introduced into the system which leads to the necessity of a very sensitive detector. It sometimes seems that non-chromatographic techniques are more suitable if sample volume is not a limitation, and thus less sensitive and less expensive detectors can be used due to the possibility of separation and pre-concentration the desired species. In addition, investment and operational costs associated with hyphenated techniques sometimes also play important roles to restrict the spread of speciation analysis as a usual task.

## 3. Detection Techniques for Speciation Analysis

The selection of detection techniques depends on the concentration level of the species presented in the sample and also the type of matrix and its composition. In terms of the detection approaches for speciation analysis, they must be selective and extremely sensitive since the species of interest usually accounts for only a small fraction of the total amount. The frequently used detection methods with high selectivity and sensitivity for As, Hg, Se and Sb species can be classified into atomic spectrometry and mass spectrometry methods.

### 3.1. Atomic Spectrometry Methods

Atomic spectrometry are subdivided into AFS, AAS, and AES (or OES), among which AFS is the most often used method, and represents a suitable alternative to the other atomic spectrometric and mass spectrometric techniques [151]. The direct coupling between HPLC and detectors will probably suffer from the interferences from the sample matrices. Therefore, when AFS is used for speciation analysis of As, Se, Sb and Hg, HG technique for As, Se, Sb, and cold vapor (CV) generation for Hg are commonly used as online post-column derivatization method to separate the analytes from sample matrices [152]. The integration of HG/CV based on the reaction of BH_4_^−^ with the acidized sample prior to detector is proved to be an effective measure to reduce interferences and background signal from the sample matrices. The detection limits lower than µg/L will be obtained using HPLC-HG-AFS for As speciation analysis [153]. However, it is noted that transition metals presented in samples will cause serious interferences in HG process. Some improving measures such as mask may be taken into consideration [77,78]. 

Although there are some reports about the applications of AFS for the speciation analysis of As, Hg, Se, and Sb in recent years [70,108,154,155,156,157,158], the development of atomic spectrometry methods has not been as fast as one would like because they usually need to be used together with HG or CV technique, during which the post-column treatment generally is required by the nature of this technique. In other word, HG is most suitable for low valence hydride-forming species, i.e., As(III), Sb(III), Hg(II) and Se(IV). However, the other inorganic or organic species can not or only present low reaction efficiency. In order to fulfill on-line and simultaneously speciation analysis, some pretreatment measures need to be adopted. For example, in order to simultaneously separate and determine organic species, on-line thermal microwave or UV irradiation prior to HG in the presence of strong oxidizing agents is developed to decompose the organic species. The latter is the most often used and inexpensive method for no cooling system is required after decomposition. For example, de Quadros et al. [159] developed a procedure for simultaneous determination of Hg(II), MeHg, EtHg by photodecomposition of organomercury compounds and reduction of Hg(II) to mercury vapor under microwave/ultraviolet (MW/UV) irradiation. Of course, some other new methods such as post-column oxidation using Fe_3_O_4_ magnetic nanoparticles [160] were also developed to on-line convert hydride generation/cold vapor generation inactive species into their active species. For example, Sun et al. [161] developed an on-line digestion device based on the nano-TiO_2_-catalyzed photo-oxidation of As species. Illuminating for 3 min can quantitatively converted As(III), As(V), MMA and DMA into As(V) at 1% K_2_S_2_O_8_ (*w*/*v*).

It is noted that the higher chemical valence of analytes would be dominant after the eluted organic compounds are oxidized either via a UV or microwave digestion process. Therefore, a pretreatment to transfer into lower valence compounds is required, that is As(V)→As(III), Sb(V)→Sb(III) and Se(VI)→Se(IV). For example, As(V) has low HG efficiency, and thus in order to improve the analytical sensitive, an acidic thiourea solution was used on-line by Yu et al. [162] and thioglycolic acid was used by Musil and Matoušek [163] to pre-reduce As(V) prior to HG reaction. However, resulting from the differences in chemical properties of each element and their associated compounds, the reactivity of these different compounds varies greatly. For example, KI can reduce As(V) and Sb(V) to As(III) and Sb(III) at room temperature, whereas it is impossible to reduce Se(VI) to Se(IV) under the same conditions.

Although there are some reports on the analysis of As, Hg, Se and Sb species by AAS [164,165,166,167] or AES [168,169,170] in recent years, AFS is described to be superior to AAS and similar to ICP-MS regarding sensitivity and linear calibration range for As and Se species in routine analysis. Analytical features such as low detection limits and wide linear calibration ranges, simplicity, lower acquisition and running costs make AFS a suitable atomic detector in speciation studies.

### 3.2. Mass Spectrometry Methods

The unquestionable advantages of ICP-MS over other species detectors are its high sensitivity and multi-element on-line detection capacity. In addition, the application of MS allows not only to obtain the information on the qualitative and quantitative contents of the sample, but also to determine the structure and molar masses of the analytes. The coupling of different separation techniques with ICP-MS has become common practice for the speciation analysis of trace As, Hg, Se and Sb [171,172]. Unlike HG/CV-AFS, no oxidation and pre-reduction steps are required for ICP-MS, unless HG is introduced in the system. 

Even though the interface of ICP-MS detector with HPLC is relatively simple, the main problem is that the mobile phase used must be compatible with detection system. Sodium or potassium phosphate buffer mobile phases often utilized in IEC are not appropriate for a MS detector. As discussed above, non-volatile buffer salts can be collected on the lenses and skimmer cones resulting in signal drift and a high maintenance level for cleaning the inner surfaces of the MS detector, and thus the use of volatile buffer systems or ones that have low residue is required [97]. Organic modifiers are often used in the mobile phase of RPC, and large volumes of organic solvent reaching the ICP probably results in an unstable plasma. In this sense, methanol is more widely used than acetonitrile for RPC mobile phases [109,111]. Meanwhile, desolvation the liquid aerosol before it reaches the ICP, simple flow splitting after the HPLC column, or the use of a small bore or microbore column is also adopted to reduce the amount of organic solvent introduced into the detector [98,173,174].

Although ICP-MS exhibits very good analytical performances for ultra-trace determination, one of its disadvantages is vulnerable to interferences with the molecular ion signals by atomic argon (Ar) and chlorine which can hinder the measurement of Se and As species. For example, when ICP-MS is used as a detector for As determination, it frequently suffers from chloride interference as ^38^Ar^37^Cl^+^, ^40^Ar^35^Cl^+^ (the *m*/*z* 75 is the same as ^75^As) are generated if samples contain high amount of chloride [175,176]. However, the introduction of ICP-MS equipped with collision/reaction cell (CRC) or dynamic reaction cell (DRC) is an effective approach to overcome these problems linked with polyatomic interferences, among which the DRC technique is introduced by using the reacting gas, such as ammonia, hydrogen, oxygen, etc. The reacting gas overcomes these interferences by atom transfer or charge transfer reactions to break the polyatomic ions into atoms or ions with different *m*/*z* [121,177,178]. For example, a DRC for spectral interferences elimination by using oxygen and ammonia as reaction gases was developed by Marcinkowska et al. [178] for multielemental speciation analysis of Cr(VI), As(III) and As(V) in water by advanced hyphenated technique HPLC/ICP-DRC-MS. Moreover, with the introduction of an additional quadrupole, ICP-tandem MS (ICP-MS/MS) provides more reaction/collision modes for interference elimination [179,180]. ICP-MS/MS can be seen as a conventional ICP-CRC-MS unit with an additional quadrupole located before the CRC. Only ions of a given *m*/*z* are allowed to enter the CRC, by which it contributes to a better control over the reactions taking place in the cell. For example, arsenic species in seafood were determined by Schmidt et al. [103] by LC-ICP-MS/MS using O_2_ as reaction gas for the conversion of ^75^As to ^75^As^16^O. ICP-MS/MS was used by Gao et al. [158] for the detection of selenium species in rice after separated by IP-RPC. Two reaction gas modes (H_2_, O_2_) and a collision mode (He) were investigated and found that H_2_ mode was the best choice for eliminating interferences and obtaining a higher signal-to-noise ratio.

MS detection allows the use of isotope dilution analysis (IDA) for speciation analysis due to its specific detection based on the ratio *m*/*z* [181]. ID-MS is considered to be an effective method offering accurate determination of elemental species with only small uncertainties. For example, post column isotope dilution with Se-78 spike was performed by Jeong et al. [182] for quantitative speciation of Se in human blood serum and urine. Internal standardization based on the species-unspecific isotope dilution analysis technique was proposed by Castillo et al. [183] to overcome the matrix effects and signal drift originated in the speciation of As in urine, by which it allows the calculation of the corrected overall species concentrations without requiring any methodological calibration. Moreover, in order to avoid the deterioration of sensitivity, accuracy, and long-term stability of system due to the direct introduction of salt- or organic-rich effluent into the instrument, a suitable interfacing technique is highly desirable to couple HPLC with ICP-MS for the detection of elemental species in complex matrices. Accordingly, the generation of volatile analytes by derivatization has been extensively and increasingly reported so as to improve the analytical sensitivity and eliminate the spectral/non-spectral interferences from the sample matrices and effluents [184,185]. As stated ahead, online hydride/vapor generation technique can effectively solve this problem. However, the species which can react with BH_4_^−^ to generation hydride/vapor are limited. For example, it is thought that Se(VI) is not reducible into hydride according to the studies on the reduction kinetics of inorganic Se species with NaBH_4_. Consequently, inorganic Se(VI) and Se(IV) analysis by HG technique must be carried out by first determining Se(IV) and then transforming Se(VI) to Se(IV) prior to the determination. Meanwhile, the stability of plasma may worsen when determined by HG-ICP-MS because online HG system delivers not only hydride vapor but also large amounts of hydrogen into the ICP. Therefore, the other alternative vapor generation techniques are developed. For example, an on-line sequential photocatalyst-assisted digestion and vaporization device was coupled between LC and ICP-MS by Tsai et al. [186] for Se speciation analysis. 

It is noted that although modern techniques using MS detection can help to obtain a better understanding of the experimental data and species identification, many analytical laboratories cannot support such instruments due to their high price and expensive maintenance. Therefore, the hyphenation of chromatography with atomic spectrometry seems to be a well substitute of mass spectrometry. 

## 4. Conclusion and Perspective

As far as the separation and detection of element speciation in environmental and biological samples are concerned, different approaches can be used based on on-line or off-line procedures. The hyphenated techniques, in which effective separation methods are coupled on-line with diverse selective and sensitive detectors, are attractive tools in the speciation analysis of As, Hg, Se and Se. Their main advantages include extremely low limits of detection and quantification, insignificant influence of interferences on the determination process, as well as very high precision and repeatability. Although powerful techniques based on MS are nowadays extensively used for the speciation analysis of trace elements, it is considered as an expensive instrument to purchase and maintain, and only few laboratories can support the high cost of such techniques. The hyphenation of chromatography to atomic spectrometry detectors especially the AFS is still liable and low-cost alternative for routine laboratories. Meanwhile, HG in conjunction with AFS detection deserves more research because it can be hyphenated easily to LC, and provides detection limits of the same order of those obtained with MS techniques. Therefore, it is predictable that HPLC coupled with HG-AFS will be promising for speciation analysis of As, Hg, Se and Sb in environmental and biological samples, and has vast potential for further development.

## Figures and Tables

**Table 1:** Main species of As, Hg, Se and Sb commonly detected in environmental and biological samples.

Element	Species	Abbreviation	Chemical Formula
As	Arsenite (arsenous acid)	As(III)	As(OH)_3_
	Arsenate (arsenic acid)	As(V)	AsO(OH)_3_
	Monomethylarsenate (Monomethylarsonic acid)	MMA	CH_3_AsO(OH)_2_
	Dimethylarsonate (Dimethylarsinic acid)	DMA	(CH_3_)_2_AsO(OH)
	Trimethylarsinic oxide	TMAO	(CH_3_)_3_AsO
	Arsenocholine	AsC	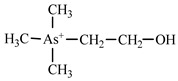
	Arsenobetaine	AsB	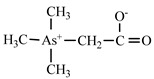
	Arsenosugars	AsS	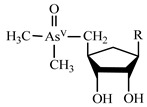
	Roxarsone	Rox	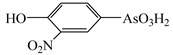
	Arsenolipids ^a^		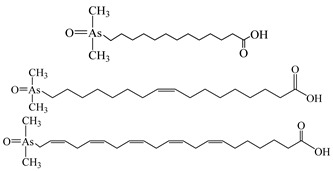
Hg	Inorganic bivalent mercury	Hg(II)	Hg^2+^
	Methylmercury	MeHg	CH_3_Hg^+^
	Dimethylmercury	DMeHg	(CH_3_)_2_Hg
	Ethylmercury	EtHg	CH_3_CH_2_Hg^+^
	Diethylmercury	DEtHg	(CH_3_CH_2_)_2_Hg
	Methylethylmercury	MEtHg	(CH_3_CH_2_)(CH_3_)Hg
	Phenylmercury	PhHg	C_6_H_5_Hg^+^
Sb	Antimonite (Antimonous acid)	Sb(III)	Sb(OH)_3_
	Antimonate (Antimonic acid)	Sb(V)	SbO(OH)_3_
	Methylantimate (Methylantimonic acid)	MMSb	CH_3_SbO(OH)_2_
	Dimethylantimate (Dimethylantimonic acid)	DMSb	(CH_3_)_2_SbO(OH)
	Trimethylantimony dichloride	TMSbCl_2_	(CH_3_)_3_SbCl_2_
Se	Selenite	Se(IV)	H_2_SeO_3_
	Selenate	Se(VI)	H_2_SeO_4_
	Selenomethionine	SeMet	CH_3_SeCH_2_CH_2_CH(NH_2_)COOH
	Selenocysteine	SeCys	HSeCH_2_CH(NH_2_)COOH
	Se-methylselenocysteine	SeMeCys	CH_3_SeCH_2_CH(NH_2_)COOH
	Selenoethionine	SeEt	CH_3_CH_2_SeCH_2_CH_2_CH(NH_2_)COOH
	Selenocystine	SeCys_2_	HOOCCH(NH_2_)CH_2_Se-SeCH_2_CH(NH_2_)COOH
	Selenohomolanthionine	SeHLan	HOOCCH(NH_2_)CH_2_CH_2_SeCH_2_CH_2_CH(NH_2_)COOH
	Trimethylselenonium ion	TMSe^+^	(CH_3_)_3_Se^+^
	Selenocyanate	SeCN^−^	N≡C―Se^−^
	Selenosugar 1	SeSug 1	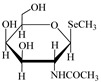
	Selenosugar 2	SeSug 2	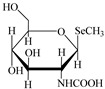
	Selenosugar 3	SeSug 3	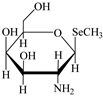

^a^ Some identified arsenic-containing fatty acids in cod-liver oil [21].

**Table 2:** Some publications of hyphenated techniques based on LC for the speciation analysis of As, Hg, Sb and Se in environmental and biological samples in recent three years.

Element	Species	Column	Detector	Matrix	Ref.
As	As(III), As(V), DMA, MMA	Hamilton PRP-X100	ICP-MS	Rice	[91]
	As(III), As(V), DMA, MMA	Agilent ZORBAX SB-Aq	ICP-MS	Cynomolgus macaques	[92]
	As(III), As(V), DMA, MMA, AsB, AsC	Dionex IonPac AS19	ICP-MS	Ophiocordyceps sinensis	[93]
	As(III), As(V), DMA, MMA, AsB	Hamilton PRP-X100	MS	Marine samples	[94]
	As(III), As(V)	Hamilton PRP-X100	ICP-MS	Spring, well, and tap water	[95]
	As(III), As(V), DMA, MMA, AsB, AsC	Dionex IonPac AS7	ICP-MS	Bones	[96]
	As(III), As(V), DMA, MMA, AsB, AsC	Dionex IonPac AS7	ICP-MS	Fish	[97]
	As(III), As(V), DMA, MMA	Homemade capillary columns	ICP-MS	Human urine	[98]
	As(III), As(V), DMA, MMA, AsB	Hamilton PRP-X10	ICP-MS/MS	Seafood	[99]
	As(III), As(V)	Hamilton PRP-X100	ICP-MS	Mexican maize tortillas	[100]
	As(III), As(V), DMA, MMA, AsB, AsC	Dionex IonPac AS19	ICP-MS	Edible Mushrooms	[101]
	As(III), As(V), DMA, MMA, AsB	Hamilton PRP-X100	HG-AFS	Seafood	[102]
	As(III), As(V), DMA, MMA, AsB	Hamilton PRP-X10	ICP-MS/MS	Seafood	[103]
	As(III), As(V), AsB	Dionex IonPac AS9-HC	ICP-MS	Water and biota samples	[104]
	As(III), As(V)	Hamilton PRP-X100	ICP-MS	Natural water	[105]
	DMA, AsB	Spheris S5SCX	ICP-MS	Fish	[106]
	As(III), As(V), DMA, MMA	Hamilton PRP-X100	ICP-MS	Environmental waters	[107]
Hg	Hg(II), MeHg, EtHg	ZORBAX SB-C18	ICP-MS	Surface water, seawater	[51]
	Hg(II), MeHg, EtHg	Athena-C18	HG-AFS	Environmental and biological samples	[108]
	Hg(II), MeHg, EtHg	ZORBAX SB-C18	ICP-MS	Sea Cucumber	[109]
	Hg(II), MeHg, EtHg	Venusil MP-C18	CV-AFS	Natural water	[110]
	Hg(II), MeHg, EtHg	PerkinElmer C8	ICP-MS	Fish oils	[111]
	Hg(II), MeHg, PhHg	Hypersil ODS2 C18	ICP-MS	Water and fish samples	[112]
	Hg(II), MeHg	CLC-ODS C18	ICP-MS	Water	[113]
	Hg(II), MeHg	CLC-ODS C18	ICP-MS	Water	[114]
	Hg(II), MeHg, EtHg	Agilent Eclipse plus C18	ICP-MS	Rice	[115]
	Hg(II), MeHg, EtHg	Synergi Hydro-RP C18	ICP-MS	Polluted sediments	[116]
Se	Se(IV), Se(VI), SeMet, SeCys	Spheris S5 SAX	ICP-MS	Chicken breast	[117]
	Se(IV), Se(VI), SeMet, SeCys	Hamilton PRP-X100	HG-AFS	Cordyceps militaris	[118]
	Se(IV), Se(VI), SeMet, SeCys, SeMeCys	StableBond C18	ICP-MS	Rice	[119]
	Se(IV), Se(VI)	ODS-3	UV-Vis	Water and biological samples	[120]
Sb	Sb(III), Sb(V)	Hamilton PRP-X100	ICP-MS	Bottled flavored drinking water	[121]
	Sb(III), Sb(V)	Hamilton PRP-X100	ICP-MS	Matrix-rich mineral water	[122]
	Sb(III), Sb(V), TMSbCl_2_	Hamilton PRP-X100	ICP-MS	Waters, juices	[123]
	Sb(III), Sb(V)	Hamilton PRP-X100	ICP-MS	Drinking water	[124]
	Sb(III), Sb(V)	Hamilton PRP-X100	HG-AFS	Soils, sediments, volcanic ashes	[125]
	Sb(III), Sb(V)	Hamilton PRP-X100	ICP-MS	Sediments, water	[126]
